# LncRNA FAS-AS1 upregulated by its genetic variation rs6586163 promotes cell apoptosis in nasopharyngeal carcinoma through regulating mitochondria function and Fas splicing

**DOI:** 10.1038/s41598-023-35502-z

**Published:** 2023-05-22

**Authors:** Zhen Guo, ZiBo Li, MengLing Zhang, MeiHua Bao, BinSheng He, XiaoLong Zhou

**Affiliations:** 1 Academician Workstation, Changsha Medical University, LeiFeng Avenue No.1501, Changsha, 410219 People’s Republic of China; 2 Hunan Key Laboratory of the Fundamental and Clinical Research on Functional Nucleic Acid, Changsha Medical University, Changsha, 410219 People’s Republic of China; 3 Hunan Key Laboratory of the Research and Development of Novel Pharmaceutical Preparations, Changsha Medical University, Changsha, 410219 People’s Republic of China; 4 School of Stomatology, Changsha Medical University, Changsha, 410219 People’s Republic of China

**Keywords:** Cancer, Cell biology, Computational biology and bioinformatics, Genetics, Molecular biology, Biomarkers, Diseases, Oncology, Risk factors

## Abstract

Nasopharyngeal carcinoma (NPC) is a common head and neck malignant with a high incidence in Southern China. Genetic aberrations play a vital role in the pathogenesis, progression and prognosis of NPC. In the present study, we elucidated the underlying mechanism of FAS-AS1 and its genetic variation rs6586163 in NPC. We demonstrated that FAS-AS1 rs6586163 variant genotype carriers were associated with lower risk of NPC (CC vs. AA, OR = 0.645, *P* = 0.006) and better overall survival (AC + CC vs. AA, HR = 0.667, *P* = 0.030). Mechanically, rs6586163 increased the transcriptional activity of FAS-AS1 and contributed to ectopic overexpression of FAS-AS1 in NPC. rs6586163 also exhibited an eQTL trait and the genes affected by rs6586163 were enriched in apoptosis related signaling pathway. FAS-AS1 was downregulated in NPC tissues and over-expression of FAS-AS1 was associated with early clinical stage and better short-term treatment efficacy for NPC patients. Overexpression of FAS-AS1 inhibited NPC cell viability and promoted cell apoptosis. GSEA analysis of RNA-seq data suggested FAS-AS1 participate in mitochondria regulation and mRNA alternative splicing. Transmission electron microscopic examination verified that the mitochondria was swelled, the mitochondrial cristae was fragmented or disappeared, and their structures were destroyed in FAS-AS1 overexpressed cells. Furthermore, we identified HSP90AA1, CS, BCL2L1, SOD2 and PPARGC1A as the top 5 hub genes of FAS-AS1 regulated genes involved in mitochondria function. We also proved FAS-AS1 could affect Fas splicing isoform sFas/mFas expression ratio, and apoptotic protein expression, thus leading to increased apoptosis. Our study provided the first evidence that FAS-AS1 and its genetic polymorphism rs6586163 triggered apoptosis in NPC, which might have a potential as new biomarkers for NPC susceptibility and prognosis.

## Introduction

Nasopharyngeal carcinoma (NPC) is one of the most common malignant tumors in otorhinolaryngological malignancies. Though the incidence of NPC is relatively low worldwide, NPC is strikingly prevalent in Southern China and Southeast Asia^1^. The age-standardized rate of NPC is 3.0/100 000 in China versus 0.4/100 000 in white populations^2^. Epstein–Barr virus (EBV) infection, genetic variations, and environmental factors have been widely regarded as the most risk factors for NPC pathogenesis^3^. Due to its non-specific symptoms and relatively complex and hidden anatomical location, NPC is often delayed in diagnosis. Statistics show that more than 70% NPC are diagnosed at advanced stages (III–IV), resulting in poorer prognosis^4^. The 5 year survival rate ranges from 95% for patients in stages I to 25.9% for patients in stage IV^5^. Therefore, early diagnosis and treatment are very important for improving the prognosis of NPC. It is of great significance to find predictive biomarkers and elucidate their molecular mechanisms for NPC susceptibility and prognosis^6^.

LncRNA is a type of RNA longer than 200 nucleotides that lacks protein-coding potential^7^. Numerous evidence has indicated lncRNAs as crucial regulators of fundamental cellular processes by interacting with DNAs, RNAs, proteins and controls gene expression at pre-transcriptional, transcriptional and post-transcriptional level^8,9^. Through these interactions, lncRNAs may influence chromatin remodeling, pre-mRNA splicing, RNA transport or nuclear retention, mRNA structure and stability, cellular compartmental distribution^10^. Accumulating evidence indicates that aberrant expression and dysfunction of lncRNAs can contribute to tumor occurrence and progression via multiple mechanisms^11–13^. However, up to date, the roles and functions of aberrantly expressed lncRNAs in the tumorigenesis and prognosis of NPC have not been fully illustrated.

FAS-AS1 was an antisense lncRNA that transcribed from the opposite strand of the intron 1 of the Fas gene, and was high expressed in heart, placenta, liver, muscles and the pancreas in normal adults^14^. FAS-AS1 is an apoptosis-related lncRNAs that known for its role in regulating Fas alternative splicing. Growing evidences have indicated FAS-AS1 was remarkably down-regulated in breast cancer, non-small lung cancer (NSCLC), gastric cancer, bladder cancer, B-cell lymphoma, and might have a potential as diagnostic biomarker^15–19^. It has been reported that FAS-AS1 inhibited cell proliferation, migration, and invasion in NSCLC cells by targeting miR-19a-5p^20^. The expression level of FAS-AS1 was significantly correlated with tumor size and lymph node metastasis in breast cancer^21^. The transcription level of FAS-AS1 was dramatically elevated by radiation exposure and etoposide induced double-strand breaks, indicating FAS-AS1 might be involved in DNA damage response^22,23^. Moreover, FAS-AS1 could inhibit cell proliferation, promote apoptosis and degrade the extracellular matrix of cartilage in osteoarthritis^24^.

Our team was committed to studying the function of aberrant lncRNAs in NPC and found the expression of FAS-AS1 was significantly down-regulated in NPC cell lines and NPC tissues for the first time. However, the expression pattern and biological function of FAS-AS1 in NPC remains largely unknown. It is well known that host genetics plays an important role in NPC tumorigenesis. We speculate the aberrant expression of FAS-AS1 might be attribute to genetic variations to a certain extent. With the advancement of high-throughput sequencing technologies, the genome landscape of human has become more and more clear^25–27^. Single-nucleotide polymorphism (SNP) is the most commonly type of genetic variants within lncRNA genes and bioinformatics approach is a cost-effective way to screening causal SNPs^28^. In the present study, we applied LncRNASNP, Ensembl, RegulomeDB, RNAfold and selected a functional SNP (rs6586163) with regulatory feature lying in FAS-AS1. Firstly, we evaluated the role of FAS-AS1 and rs6586163 in NPC susceptibility and prognosis. Secondly, we explored how rs6586163 affect FAS-AS1 function. Thirdly, we unveiled the function and underlying mechanisms of FAS-AS1 in apoptosis in NPC. Collectively, we suggested that FAS-AS1 rs6586163 might contribute to ectopic expression of FAS-AS1 through increasing its transcriptional activity and overexpression of FAS-AS1 inhibited NPC cell viability and promoted cell apoptosis via regulating mitochondria function and suppressing sFas expression. These results indicated FAS-AS1 and its genetic polymorphism rs6586163 might have a protective role in NPC, and might serve as potential predictive genetic predisposition biomarkers for NPC.

## Materials and methods

### Cell lines and cell culture

The human immortalized normal nasopharyngeal epithelial cell line NP69 and NPC cell lines (HNE1, HONE1) were purchased from the Advanced Research Center of Central South University (Changsha, Hunan, China). NPC cells were incubated in RPMI-1640 medium (Invitrogen, Carlsbad, CA, USA) and NP69 cells were maintained in K-SFM medium (Invitrogen, Carlsbad, CA, USA), which were supplemented with 10% fetal bovine serum (Gibco; Thermo Fisher Scientific, Inc.) and 1% penicillin–streptomycin (Gibco; Thermo Fisher Scientific, Inc.) in an incubator at 37 °C with 5% CO_2_.

### Clinical specimens

684 newly diagnosed NPC patients and 823 cancer-free healthy control subjects were recruited from Hunan Provincial Cancer Hospital and Xiangya Hospital between 2015 and 2016. 3 ml of peripheral venous blood of each participant was collected and stored at − 80 °C. 92 pathologically confirmed NPC tissue biopsies and 10 chronic inflammation of nasopharyngeal mucosa tissue biopsies were collected from Hunan Provincial Cancer Hospital between 2015 and 2016. None of the patients have received any anticancer treatments before joining in the trial. Patients who had other malignancy or concomitant malignant diseases were excluded. All of the patients received intensity-modulated radiotherapy (IMRT) technique and were treated with platinum-based induction chemotherapy (IC) plus concurrent chemoradiotherapy (CCRT) regimen. The short-term efficacy of chemoradiotherapy was evaluated using the Response Evaluation Criteria in Solid Tumor (RECIST) guidelines according to the magnetic resonance imaging (MRI) 3 months after treatment. The survival status of patients was followed up by telephone once every 3 months.

This study was approved by the Independent Ethical Committee of Institute of Clinical Pharmacology, Central South University (CTXY-140007-2). All the participants signed informed consent at the time of enrollment. This study was performed in accordance with the Declaration of Helsinki.

### DNA extraction and genotyping

Genomic DNA was extracted from peripheral blood lymphocytes and NPC tissues using the QIAamp DNA Mini and Blood Mini Kit (Qiagen Inc., Valencia, CA, USA). The concentrations and purity of DNA were measured using NanoDrop TM 1000 spectrophotometer. Genotype of the candidate SNP was determined by Sequenom MassARRAY iPLEX (Sequenom, Inc., San Diego, CA, USA). The call rate threshold was at least 95%.

### RNA isolation and real-time PCR

Total RNA was isolated from tissues or cells using RNAiso Plus reagent (Takara, Japan) and reverse-transcribed to complementary DNA using a PrimeScript RT Reagent kit (Takara, Japan). qPCR was performed using SYBR® Premix ExTaq™ (Takara, Japan) according to the manufacturer's instructions. The primer sequences were as follows: GAPDH-F: 5′-ACAACTTTGGTATCGTGGAAGG-3′, GAPDH-R: 5′- GCCATCACGCCACAGTTTC-3′; FAS-AS1-F: 5′-GCGTGTGTGTGTGTATTCTTTC-3’, FAS-AS1-R: 5′-AGCTTGGAGCTATGCTTGTT-3′; FAS-F: 5′-CAAGGGATTGGAATTGAGGA-3′, FAS-R: 5′-CTGGAGGACAGGGCTTATGG-3′. The 2^−ΔΔCt^ method was used to calculate the relative expression levels of target mRNA.

### Bioinformatics analysis

LncRNASNP and RegulomeDB was used to select functional SNP in FAS-AS1 and identify DNA features and regulatory elements at rs6586163. In silico prediction of the folding structure and mountain plot of FAS-AS1 rs6586163 wild type and mutant type were computed by RNAfold WebServer. The eQTL data of rs6586163 was downloaded from Ensembl. GO enrichment analysis was performed using the online The Gene Ontology Resource (http://geneontology.org/). KM Plotter (http://kmplot.com/analysis/index.php?p=background) was applied to evaluate the prognosis role of FAS-AS1 in other types of cancers. The PPI network of FAS-AS1 regulated mitochondria related genes were constructed with String database and analyzed by Cytoscape 3.9.1 software. The hub gene of the network was analyzed by cytoHubba tools.

### Plasmid construction and cell transfection

The full length of human FAS-AS1 cDNA was directly synthesized by Genechem Company (Genechem, Shanghai, China) and cloned into pcDNA 3.1 plasmid to construct the FAS-AS1 overexpression vector. A length of 500 nt base covering wild-type of FAS-AS1 rs6586163 was directly synthesized by Genechem (Genechem, Shanghai, China) and cloned into KpnI/XhoI site of GV148 vector to get the wild-type luciferase reporter vector (WT). Site-directed mutagenesis at rs6586163 was utilized to get the mutant-type luciferase reporter vector (MUT). DNA sequencing was utilized to confirm the sequence of these vectors. Transfection was carried out using Lipofectamine 3000 reagent (Invitrogen, Carlsbad, CA, USA).

### Dual luciferase reporter assay

The cells were co-transfected with Renilla vector and FAS-AS1-WT vector or FAS-AS1-MUT vector or control vector for 48 h. Then, cells were harvested and luciferase activities were assayed using the Dual Luciferase Reporter Assay Kit (Promega, Madison, WI, USA) according to the manufacturer’s instructions. Firefly luciferase activity was normalized to Renilla luciferase activity in order to correct the transfection efficiency.

### RNA-seq

HNE1 cells were transfected with FAS-AS1 overexpression vector or control vector for 24 h. Then, total RNA was extracted and the libraries were constructed using VAHTS Universal V6 RNA-seq Library Prep Kit. The transcriptome was sequenced by OE Biotech Co., Ltd. (Shanghai, China) using an Ilumina Novaseq 6000 platform. Q value < 0.05 and fold change > 2 was set as the threshold for significantly differential expression gene (DEGs). Gene Set Enrichment Analysis (GSEA) was performed using GSEA software. The heatmap and KEGG enrichment analysis was generated by online platform for data analysis and visualization named Metascape (https://metascape.org/gp/index.html#/main/step1) and Bioinformatics (http://www.bioinformatics.com.cn/).

### CCK-8 cell viability assay

The cells transfected with FAS-AS1 overexpression vector or control vector were seeded into 96-well plates at a density of 2000 cells/well. After being cultured for 24 h, 48 h, 72 h, 96 h, the cell culture medium was removed, and 100 µL cell culture medium containing 10 µL CCK-8 reagent (BioGLP, Montclair, CA, USA) was added into each well. Then, the cells were incubated in the dark for 1 h at 37 °C. The absorbance was measured at 450 nm using a spectrophotometer for analyzing cell viability.

### CellTiter luminescent cell viability assay

NPC cells were seeded into 96-well plate and transfected with FAS-AS1 overexpression vector or control vector. The next day, take out the cell culture plate and balance it at room temperature for 10 min. Add 100 μL CellTiter-Lumi to each well and shake it at room temperature for 2 min to lysis cell. Then, incubate the cells at room temperature for 10 min to stabilize the luminous signal. Chemiluminescence was detected by a multifunctional microplate reader.

### Cell cycle progression

The cells were transfected with FAS-AS1 overexpression vector or control vector for 24 h. Then, they were carefully harvested, resuspended in pre-cooled 70% ethanol, and stored at − 20 °C overnight. After centrifugation and washing by PBS, the cells were resuspended in 0.5 mL propidium staining buffer (Beyotime, China) containing RNase and incubated at 37 °C for 30 min. Finally, cell cycle analysis was performed using BD FACSDiva and FlowJo software.

### Mito-Tracker red CMXRos staining

NPC cells were seeded into 24-well plate and transfected with FAS-AS1 overexpression vector or control vector. 24 h after transfection, remove the cell culture medium, add Mito Tracker Red CMXRos working solution, and incubate at 37 °C for 15–30 min. Then, remove Mito Tracker Red CMXRos working solution, and add fresh cell culture medium. Observe and capture representative images with fluorescence microscope.

### Annexin V-FITC cell apoptosis assay

NPC cells were seeded into 6-well plate and transfected with FAS-AS1 overexpression vector or control vector for 48 h. Then, the cells were harvested and resuspended in the binding buffer and stained with FITC-Annexin V (AV) and propidium iodide (PI) according to the protocols (Beyotime, China). Cell apoptosis was immediately analyzed with a flow cytometry (BD Biosciences, San Jose, CA, USA).

### Hoechst staining

NPC cells were seeded into 24-well plate and transfected with FAS-AS1 overexpression vector or control vector. 24 h after transfection, the cells were washed by PBS, fixed by paraformaldehyde for 10 min, and stained with Hoechst33342 (Beyotime, China) for 15 min in darkness. Then, remove the staining solution and wash the cells with PBS. The EVOS M7000 Imaging System was used to capture representative images.

### SYTO-9/PI staining

NPC cells were seeded into 24-well plate and transfected with FAS-AS1 overexpression vector or control vector. 24 h after transfection, the cells were washed by PBS and co-stained with PI and SYTO 9 (Invitrogen, Carlsbad, CA, USA) for 15 min in darkness. SYTO 9 can enter all cells regardless of their membrane integrity, bind to DNA and RNA and emit green fluorescence while PI can only enter cells with compromised membranes, bind to DNA and RNA and emit a red fluorescent signal. Then, remove the staining solution and wash the cells with PBS. The EVOS M7000 Imaging System was used to capture representative images.

### Transmission electron microscopy (TEM)

The morphology and ultrastructure of mitochondrial in NPC cells were captured by TEM. Cells were fixed in TEM fixative at 4 °C for 4 h and pre-embedded in 1% agarose. The samples were post fixed with 1% OsO_4_ in 0.1 MPB (pH 7.4) for 2 h at room temperature and dehydrated using a gradient series of ethyl alcohol. Samples were then embedded in Embed 812 resin and cut to 60–80 nm thin on the ultra microtome. The sections were stained with 2% uranyl acetate and lead citrate and observed using a transmission electron microscope (Hitachi, Japan).

### Western blotting

NPC cells were transfected with FAS-AS1 overexpression vector or control vector using Lipofectamine 3000. After 48 h, the cells were collected and lysed in RIPA buffer (Beyotime, China) with 1% PMSF and phosphatase inhibitor cocktail on ice for 30 min. The concentration of protein was detected by BCA kit (Beyotime, China). A total of 10 μg protein was loaded into a SDS-PAGE gel and separated by electrophoresis. The blots were transferred to PVDF membranes at 250 mA for 90 min. The membranes were blocked with 5% skimmed milk for 1 h and incubated with primary antibodies against cleaved caspase 3 (cat: A11021, ABclonal), bax (cat: A0207, ABclonal), bcl2 (cat: A19693, ABclonal) and β-Actin (cat: AC026, ABclonal) at 4 °C overnight. Then, the blots were incubated with secondary antibodies at room temperature for 1 h. After washing three times with TBST, the blots were detected by ECL kit and visualized by ChemiDoc MP Imaging System.

### Statistical analysis

Multivariate logistic regression analysis was used to assess the association between the selected SNP and NPC susceptibility. Kaplan–Meier analysis and Cox proportional hazards regression analysis were applied to evaluate the overall survival of NPC patients. All in vitro experiments were performed at least three times and the data were represented as means ± standard deviations. A two-tailed Student’s t-test was used to compare significant difference between two groups. Statistical analyses were carried out using SPSS software (SPSS, Chicago, IL, USA) and GraphPad Prism software (GraphPad, San Diego, CA, USA). *P* value < 0.05 was considered as statistically significant, ns means no significance, *P < 0.05, **P < 0.01.

## Results

### FAS-AS1 rs6586163 was associated with the occurrence and survival of NPC

**Figure 1:**
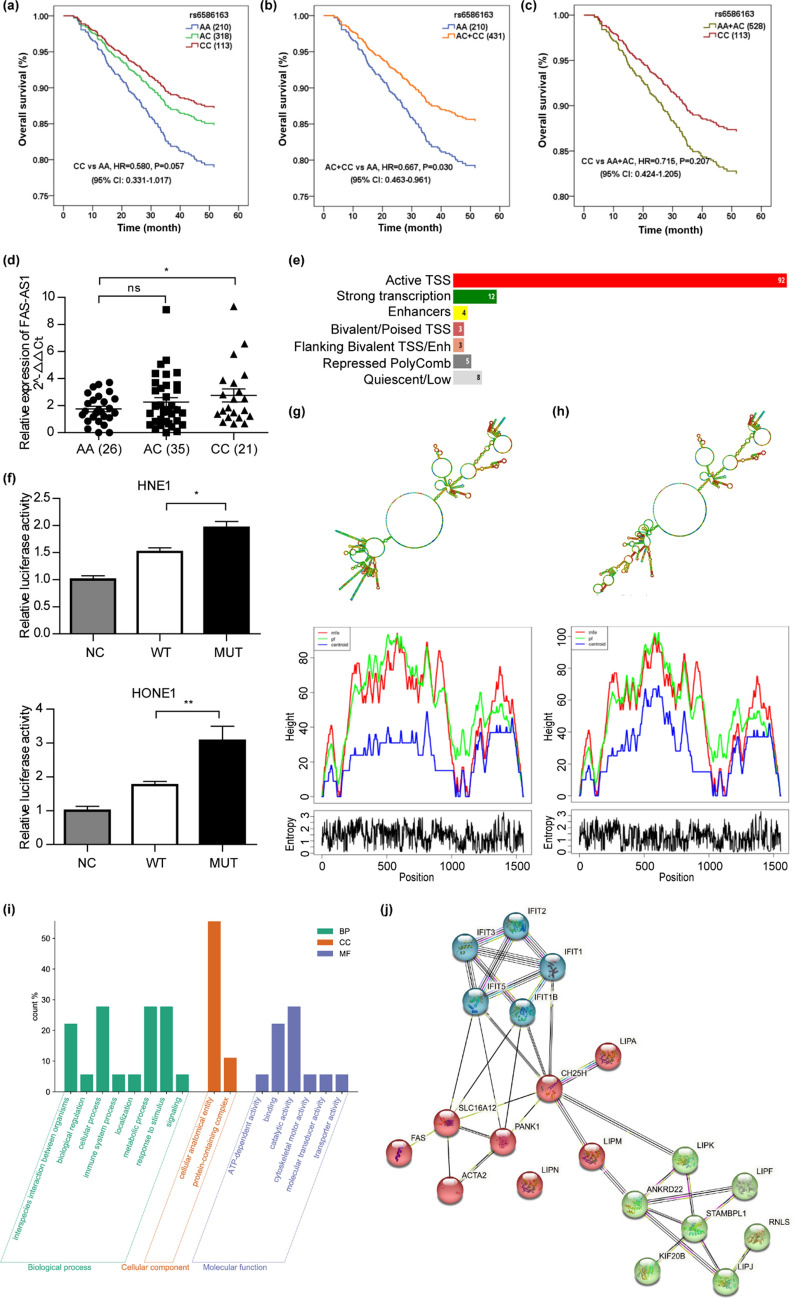
The role of FAS-AS1 rs6586163 in NPC. (**a**) The association of FAS-AS1 rs6586163 and overall survival of NPC patients in co-dominant model. (**b**) The association of FAS-AS1 rs6586163 and overall survival of NPC patients in dominant model. (**c**) The association of FAS-AS1 rs6586163 and overall survival of NPC patients in recessive model. (**d**) Relative expression of FAS-AS1 in NPC tissues with different genotypes of rs6586163. (**e**) The chromatin state at rs6586163. (**f**) Relative luciferase activity in NPC cells that transfected with rs6586163 C allele (MUT) reporter plasmid or rs6586163 A allele (WT) reporter plasmid. (**g**) The local centroid secondary structure (up) and the mountain plot indicating the positional entropy for each position (down) of the wild type FAS-AS1. (**h**) The local centroid secondary structure (up) and the mountain plot indicating the positional entropy for each position (down) of rs6586163 mutant FAS-AS1. (**i**) GO enrichment analysis of genes affected by FAS-AS1 rs6586163. (**j**) PPI network was constructed of the genes regulated by rs6586163. *P < 0.05, **P < 0.01.

The detailed clinical information of the enrolled NPC patients and control subjects was not shown in the present study, as it has been previously reported by our team in other articles^29^. The genotyping data of rs6586163 was listed in Supplementary table 2 and genotype frequency of rs6586163 was in accordance with Hardy–Weinberg equilibrium. The allele frequency of rs6586163 A allele was 0.5109 in 1000 Genomes-East Asian population and 0.5244 in our control cohort, while it increased to 0.5772 in our NPC cohort. To assess the association between rs6586163 (A > C) and the risk of NPC, we conducted multivariate logistic regression analysis that adjusted for age, gender, BMI, smoking status, and drinking status. Our results revealed that rs6586163 CC genotype carriers had a 35.5% lower risk of NPC compared with AA genotype carriers (CC vs. AA, OR = 0.645, *P* = 0.006) (Table 1). Cox proportional hazards regression analysis was applied to evaluate the effect of rs6586163 on survival in co-dominant, dominant and recessive genetic models. The mean follow-up time was 49.27 months (range, 3.5–60 months). We demonstrated that the CC genotypes was associated with longer overall survival, though the *P* value was not significant (CC vs. AA, HR = 0.580, *P* = 0.057). In the dominant model, patients with AC + CC genotypes showed a significantly better overall survival than AA genotype (AC + CC vs. AA, HR = 0.667, *P* = 0.030). The median survival time was 53.602 months for AA + CC genotypes carriers and 50.905 months for AA genotype carriers (Fig. 1a–c).

**Table 1:** Association between rs6586163 and NPC susceptibility.

Genotype	Control	Case	OR (95% CI)	P*
rs6586163(A > C)				
AA	224 (27.4)	223 (32.8)	1.00 (reference)	
AC	410 (50.1)	339 (49.9)	0.839 (0.654–1.076)	0.166
CC	184 (22.5)	118 (17.3)	0.645 (0.472–0.883)	**0.006**
AC + CC versus AA			0.779 (0.616–0.985)	**0.037**
CC versus AC + AA			0.720 (0.548–0.945)	**0.018**

*Adjusted by age, gender, BMI, smoking status, and drinking status.

Significant values are in bold.

### rs6586163 affects the expression and secondary structure of FAS-AS1

We wondered how rs6586163 exert its function in NPC occurrence and prognosis. On the one hand, we detected the expression level of FAS-AS1 with different genotype of rs6586163 in NPC tissues. Our result showed that the expression of FAS-AS1 was significantly higher in CC genotype carriers than AA genotype carriers (Fig. 1d). This is in line with our expectation considering the regulatory feature of rs6586163. We used RegulomeDB (https://www.regulomedb.org/regulome-search) to identify DNA features and regulatory elements in non-coding regions. As shown in Fig. 1e, the chromatin state at rs6586163 (chr10:90752017-90752018) was enriched with active transcription start sites (TSS), strong transcription and enhancers in many cell lines, indicating its role in regulating transcription. So, we performed dual luciferase reporter assay to detect the transcriptional ability of FAS-AS1 with different rs6586163 allele. We found the relative luciferase activity was significantly higher in NPC cells transfected with the rs6586163 C allele (MUT) reporter plasmid than rs6586163 A allele (WT) reporter plasmid (Fig. 1f). Collectively, we proposed that rs6586163 might increase the expression of FAS-AS1 by enhancing the transcriptional activity of FAS-AS1.

On the other hand, we conducted in silico analysis to predict the effect of rs6586163 on the folding structure of FAS-AS1. As shown in Fig. 1g and h, the local centroid secondary structure was greatly changed following the rs6586163 A to C alteration and the minimum free energy was decreased from − 214.66 kcal/mol to -259.96 kcal/mol. Given that the secondary structure was vital for lncRNA to interact with other proteins/RNAs, we suggested rs6586163 might affect the function of FAS-AS1 by changing its secondary structure to some extent.

### GO enrichment analysis of genes affected by rs6586163

Furthermore, data from Ensembl (http://asia.ensembl.org/) indicated rs6586163 might have an eQTL trait and was correlated with expression of 36 genes in 84 tissues (Supplementary table 1). We performed GO functional enrichment analysis to investigate the role of these genes affected by rs6586163. For the molecular function (MF) category, the genes were predominantly enriched in binding and catalytic activity. For the cellular component (CC) category, the genes were enriched in cellular anatomical entity. For the biological process (BP) category, the genes were involved in response to stimulus, cellular process and metabolic process (Fig. 1i). These genes affected by rs6586163 might participate in the regulation of FAS signaling pathway, p53 pathway, apoptosis signaling pathway, and Wnt signaling pathway, all of which were vital in controlling cell apoptosis in cancer. We also constructed the protein–protein interaction (PPI) network of these genes by String (http://string-db.org/), and the sub-networks were involved in lipid metabolism, antiviral defense and interferon alpha/beta signaling (Fig. 1j). Together, the above results indicated that FAS-AS1 rs6586163 might regulate the expression of FAS-AS1 and apoptosis signaling pathway, thus contributing to NPC occurrence and prognosis.

### FAS-AS1 was downregulated in NPC and correlated with prognosis of various cancers

**Figure 2:**
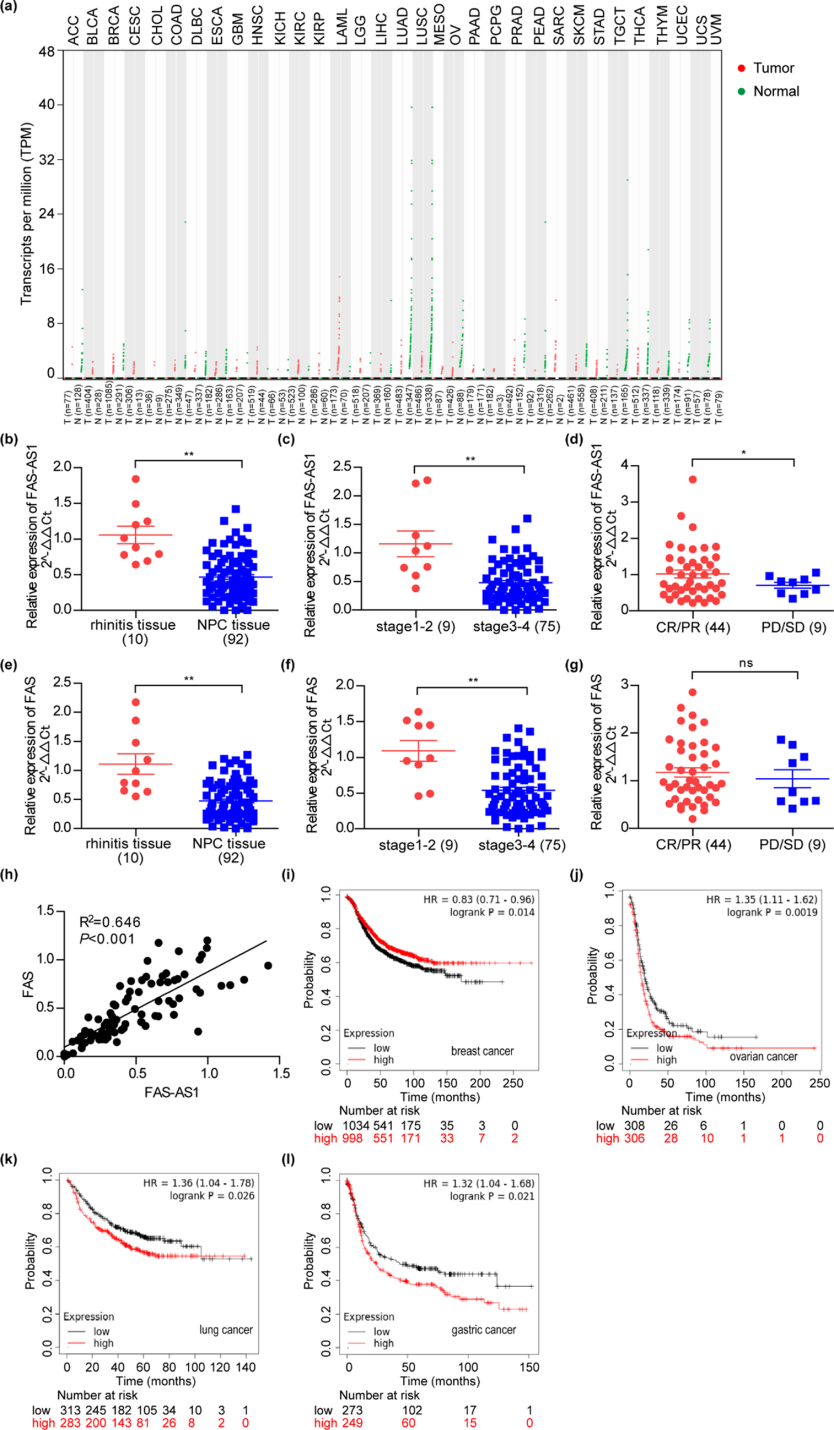
FAS-AS1 was downregulated in NPC and correlated with prognosis of various cancers. (**a**) The gene expression profile of FAS-AS1 across all tumor samples and paired normal tissues. Each dots represent expression of samples. (**b**) Relative expression of FAS-AS1 in NPC tissues and rhinitis tissues were examined by RT-qPCR. (**c**) The expression level of FAS-AS1 was associated with clinical stage of NPC patients. (**d**) The expression level of FAS-AS1 was associated with short-term treatment efficacy of NPC patients. (**e**) Relative expression of FAS in NPC tissues and rhinitis tissues were examined by RT-qPCR. (**f**) The expression level of FAS was associated with clinical stage of NPC patients. (**g**) The expression level of FAS was not associated with short-term treatment efficacy of NPC patients. (**h**) Correlation analysis of FAS-AS1 and FAS in NPC tissues. (**i**–**l**) FAS-AS1 was associated with survival in breast cancer (**i**), ovarian cancer (**j**), lung cancer (**k**), and gastric cancer (**l**). *P < 0.05, **P < 0.01.

To better understanding the role of FAS-AS1 in tumors, we investigated the expression profile of FAS-AS1 across 33 types of tumor samples and paired normal tissues by GEPIA. As shown in Fig. 2a, the expression pattern of FAS-AS1 was inconsistent in different cancers, as it might be significantly upregulated in acute myeloid leukemia (LAML) while downregulated in lung adenocarcinoma (LUAD) and lung squamous cell carcinoma (LUSC). Up to date, no one has reported the expression feature of FAS-AS1 in NPC. To investigate the role of FAS-AS1 in NPC pathogenesis and prognosis, we examined the expression of FAS-AS1 in 92 NPC tissues and 10 rhinitis tissues by qRT-PCR and evaluated the short-term treatment efficacy of NPC patients. Compared with rhinitis tissues, the expression level of FAS-AS1 was significantly down-regulated (0.466-fold change) in NPC tissues (*P* < 0.01) (Fig. 2b). Moreover, the expression of FAS-AS1 also correlated with clinical stage of NPC patients. Patients at late clinical stage (III-IV) showed a 0.479-fold decreased level of FAS-AS1 (Fig. 2c). Over-expression of FAS-AS1 was associated with better short-term treatment efficacy, suggesting FAS-AS1 might be a potential protective factor in NPC prognosis (Fig. 2d).

Considering the role of FAS-AS1 in regulating FAS splicing, we also detected the expression of FAS in NPC tissues. The expression pattern of FAS was similar with FAS-AS1 in NPC. As shown in Fig. 2e and f, FAS was down-regulated in NPC tissues and lower expression of FAS was correlated with late clinical stage. However, the relationship between FAS and short-term treatment efficacy was not remarkable (Fig. 2g). The Pearson correlation analysis revealed the expression of FAS was positively associated with FAS-AS1 in NPC tissues (R^2^ = 0.646, *P* < 0.001) (Fig. 2h).

We applied KM Plotter (http://kmplot.com/analysis/index.php?p=background) to further evaluate the role of FAS-AS1 in other types of cancers. The data indicated overexpression of FAS-AS1 was associated with better progression-free survival (PFS) in breast cancer, as the median survival time increased from 34.8 months to 44 months (Fig. 2i). This suggested FAS-AS1 might have a protective function in breast cancer, which was similar with our result in NPC. However, the result was reverse in ovarian cancer, lung cancer, and gastric cancer, as overexpression of FAS-AS1 showed a poorer PFS (Fig. 2j–l). This is quite interesting and indicates FAS-AS1 might have dual effect in different cancers that needs further exploration.

### FAS-AS1 inhibits cell viability and promotes apoptosis

**Figure 3:**
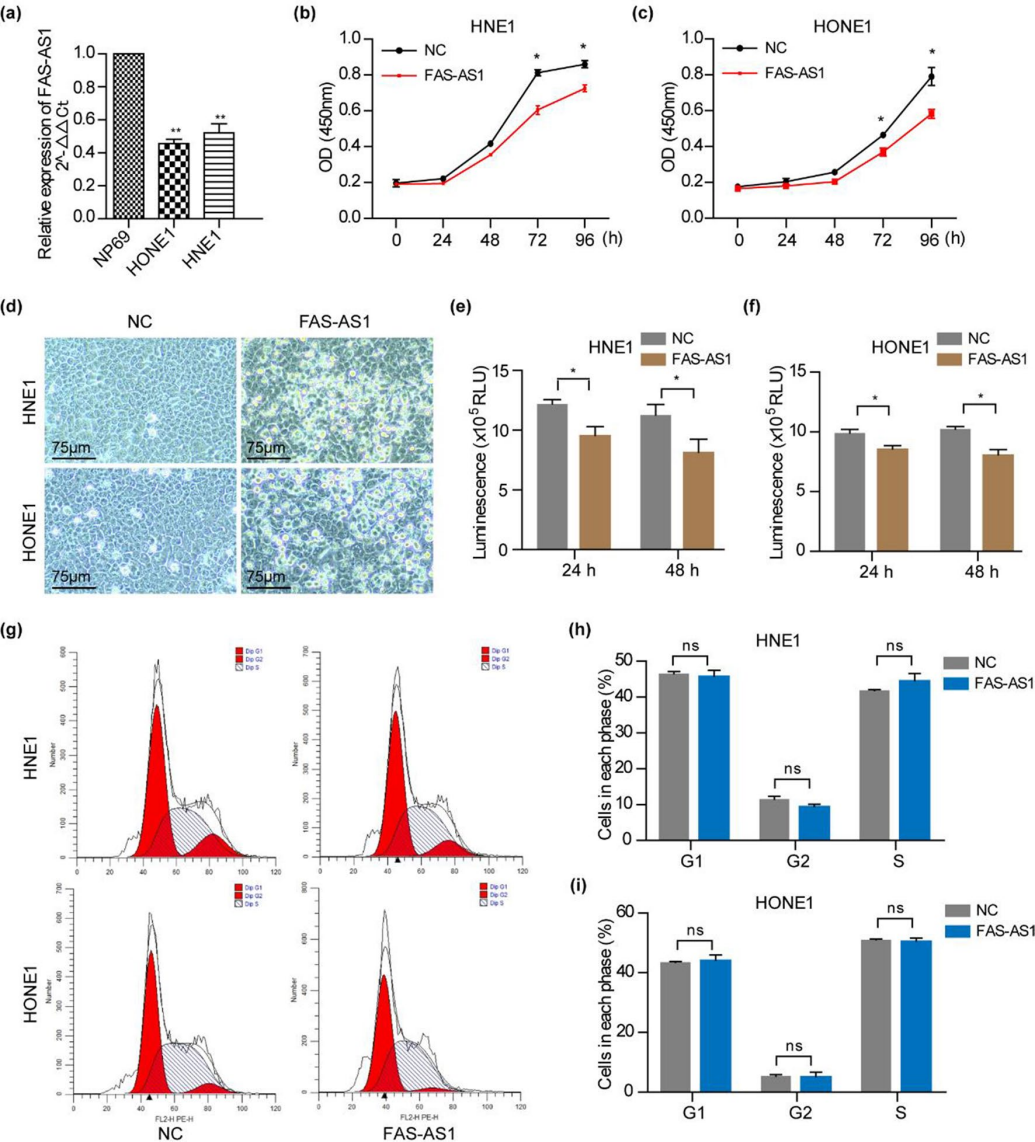
FAS-AS1 inhibits NPC cell viability. (**a**) Relative expression of FAS-AS1 in NPC cell lines (HNE1, HONE1) and normal nasopharyngeal epithelial cell line NP69. (**b**) Cell viability was measured by CCK-8 assay in HNE1 cells. (**c**) Cell viability was measured by CCK-8 assay in HONE1 cells. (**d**) The morphology of HNE1 and HONE1 cells 24 h after transfection with FAS-AS1 vector or control vector. (**e**) Cell viability was measured by CellTiter luminescent cell viability assay in HNE1 cells. (**f**) Cell viability was measured by CellTiter luminescent cell viability assay in HONE1 cells. (**g**) Cell cycle was determined by flow cytometry 24 h after transfection with FAS-AS1 vector or control vector. (**h**) Quantitative analysis of cells in each cell cycle phase in HNE1 cells. (**i**) Quantitative analysis of cells in each cell cycle phase in HONE1 cells.

**Figure 4:**
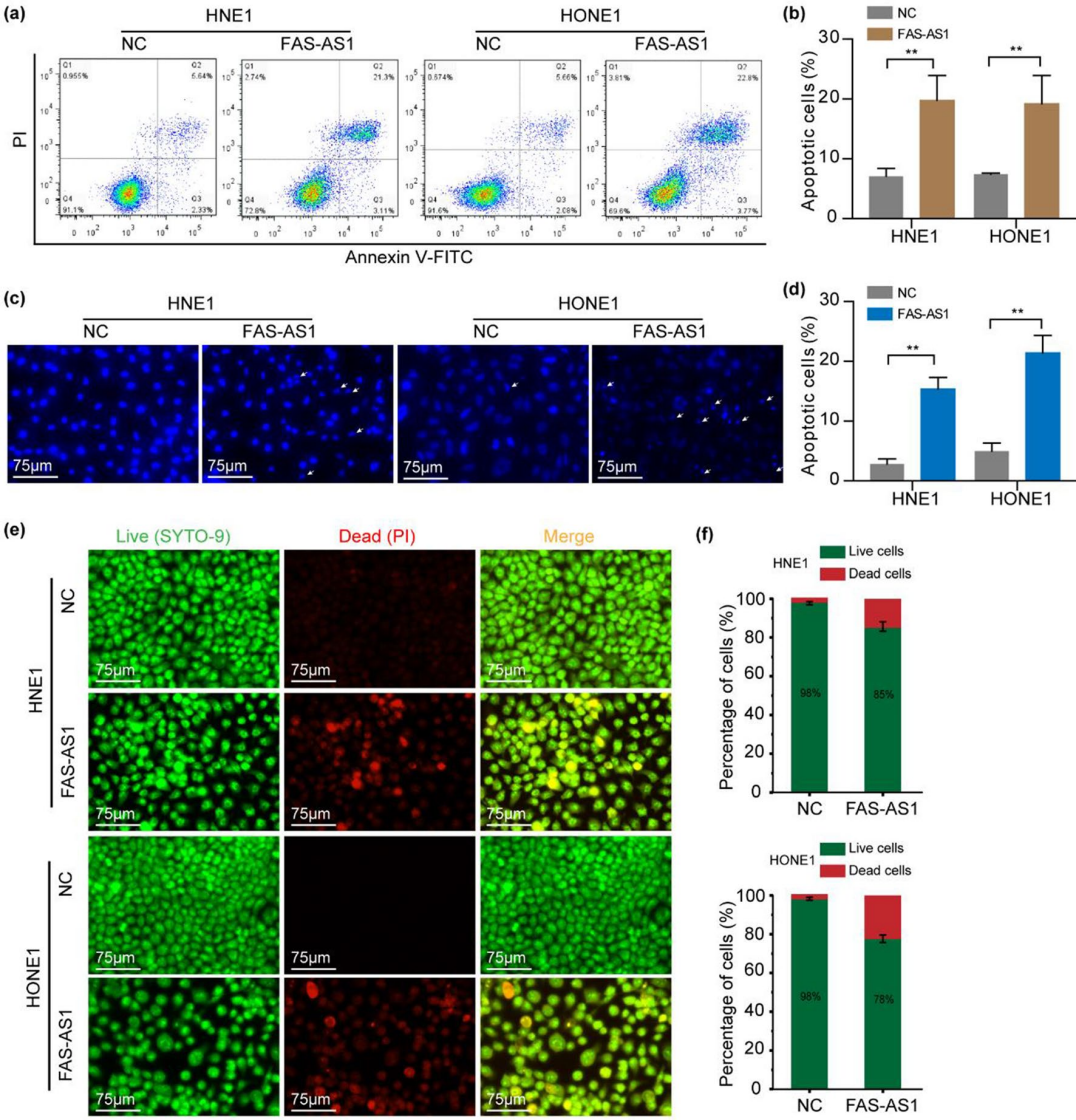
FAS-AS1 promotes NPC cell apoptosis. (**a**) Representative picture of Annexin V-FITC cell apoptosis assay in FAS-AS1 overexpression group or control group. (**b**) Quantitative analysis of the apoptotic percentage of Annexin V-FITC cell apoptosis assay. (**c**) Representative image of Hoechst staining in FAS-AS1 overexpression group or control group. (**d**) Quantitative analysis of the apoptotic percentage of Hoechst staining. (**e**) Representative image of SYTO-9/PI staining in FAS-AS1 overexpression group or control group. (**f**) Quantitative analysis of the apoptotic percentage of SYTO-9/PI staining. *P < 0.05, **P < 0.01.

To characterize the function of FAS-AS1 in NPC cells, we detected its expression in human NPC cell lines and observed a significant downregulation of FAS-AS1 in NPC cell lines (HNE1, HONE1) compared with normal nasopharyngeal epithelial cell line NP69 (Fig. 3a). In order to gain insight into the molecular mechanisms of FAS-AS1 on NPC cell growth and apoptosis, we conducted function gain study by transfecting FAS-AS1 overexpression plasmid in HNE1 and HONE1 cells. We found ectopic overexpression of FAS-AS1 inhibited NPC cell viability in a time dependent pattern (Fig. 3b,c). Moreover, we observed the cell morphology changed significantly, as the adherent cells shrinking, rounded and felling off after transfection with FAS-AS1 (Fig. 3d). CellTiter luminescent cell viability assay showed the intracellular ATP content was decreased in FAS-AS1 overexpressed cells (Fig. 3e,f). However, Flow cytometry analysis demonstrated the cell cycle progression was not affected by FAS-AS1 (Fig. 3g-i). Results from Annexin V-FITC cell apoptosis assay (Fig. 4a,b), Hoechst staining (Fig. 4c,d) and SYTO-9/PI staining assay (Fig. 4e,f) also indicated that the apoptotic cells was significantly increased in FAS-AS1 overexpressed cells.

### GSEA analysis of gene sets regulated by FAS-AS1

**Figure 5:**
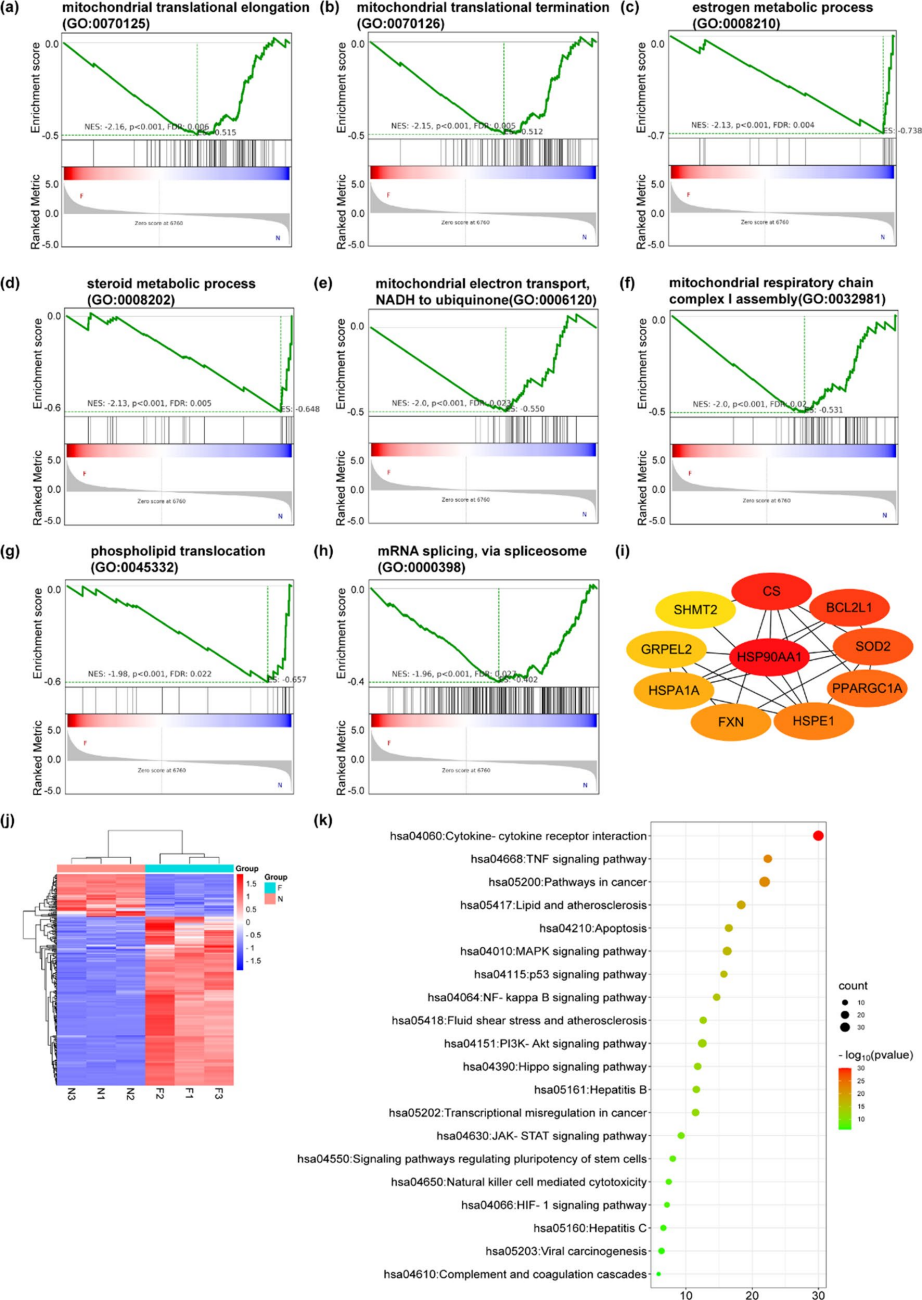
GSEA analysis of gene sets regulated by FAS-AS1. (**a**–**h**) The top eight biological process terms enriched by GSEA analysis. (**i**) The hub genes of mitochondria related DEGs regulated by FAS-AS1. (**j**) The heatmap of apoptosis related genes in FAS-AS1 overexpression group and control group was generated by an online platform named Bioinformatics (http://www.bioinformatics.com.cn/). (**k**) KEGG enrichment analysis of the apoptosis related DEGs was analyzed by Metascape (https://metascape.org/gp/index.html#/main/step1) and visualized by Bioinformatics.

In order to explore the biological signaling pathway regulated by FAS-AS1 in NPC, RNA-seq was performed in the FAS-AS1 overexpressed group and control group. Then, we conducted GSEA analysis to identify the functional gene sets. As exhibited in Fig. 5a–h, the top eight biological process terms were enrichment in mitochondrial translational elongation (GO:0070125), mitochondrial translational termination (GO:0070126), steroid metabolic process (GO:0008202), estrogen metabolic process (GO:0008210), mitochondrial electron transport (GO:0006120), mitochondrial respiratory chain complex I assembly (GO:0032981), phospholipid translocation (GO:0045332), and mRNA splicing (GO:0000398). We noticed that most of the eight terms were associated with mitochondrial, indicating FAS-AS1 might play an important role in regulating the function of mitochondrial. We then put the mitochondrial related differentially expressed genes (DEGs) into the String database to build a PPI network and analyzed the hub genes in the network using cytohubba. We identified heat shock protein 90 alpha family class A member 1 (HSP90AA1), citrate synthase (CS), BCL2 like 1 (BCL2L1), superoxide dismutase 2 (SOD2), PPARG coactivator 1 alpha (PPARGC1A), heat shock protein family E (Hsp10) member 1 (HSPE1), frataxin (FXN), heat shock protein family A member 1A (HSPA1A), GrpE like 2, mitochondrial (GRPEL2) and serine hydroxymethyltransferase 2 (SHMT2) as the top 10 hub genes of the network (Fig. 5i). These proteins might be the main potential targets of FAS-AS1 in mitochondrial dysfunction. The functions of these proteins will be discussed in depth in the Discussion section.

As we all know, mitochondrial dysfunction is closely related to cell apoptosis. We extracted the DEGs associated with apoptosis and conducted enrichment analysis. The heatmap revealed these genes could clearly distinguish the FAS-AS1 overexpressed group and control group (Fig. 5j). KEGG enrichment analysis demonstrated these apoptosis related DEGs were mainly enriched in cytokine-cytokine receptor interaction, TNF signaling pathway, pathways in cancer, lipid and atherosclerosis, and apoptosis (Fig. 5k).

### FAS-AS1 promotes apoptosis through regulating mitochondria function and Fas splicing

**Figure 6:**
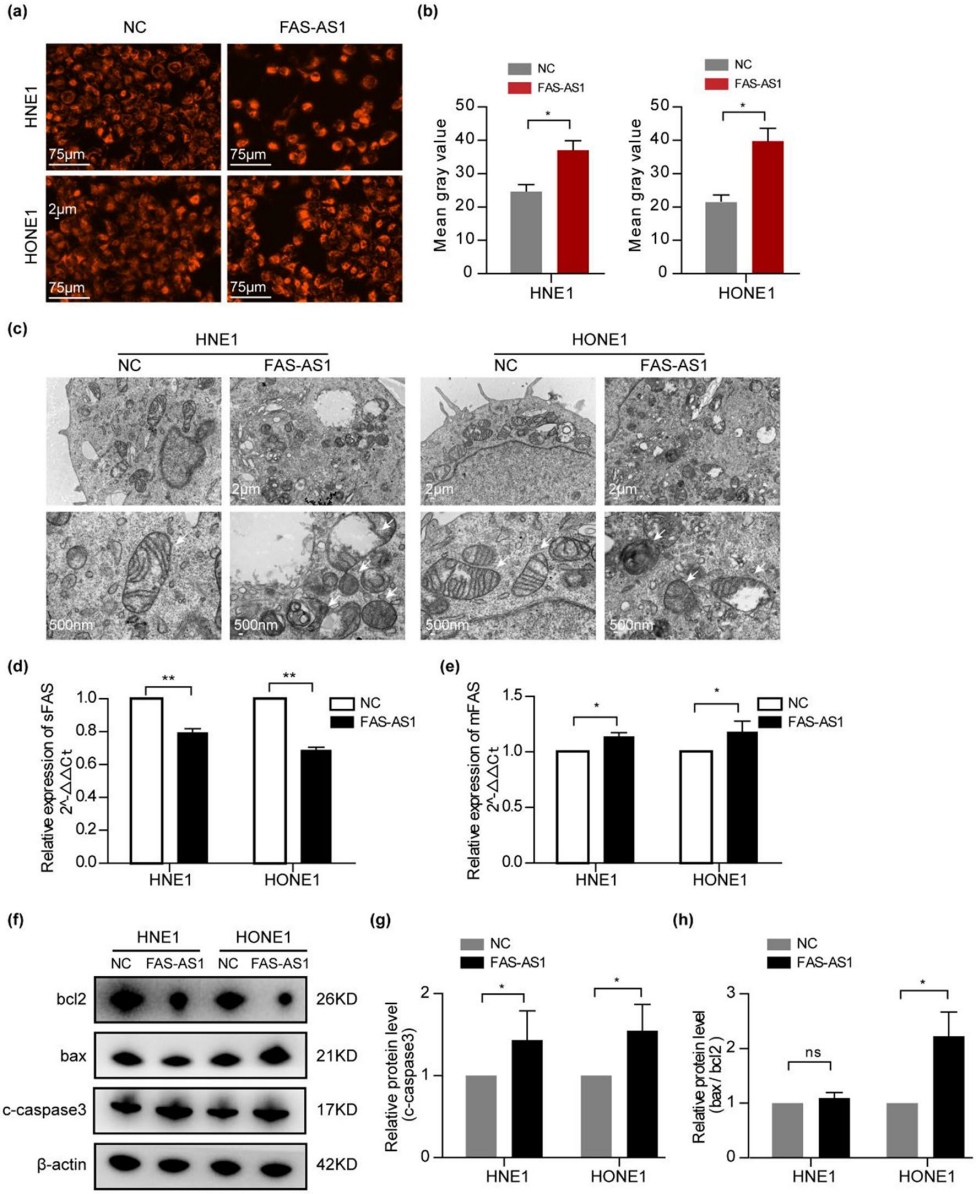
FAS-AS1 promotes apoptosis through regulating mitochondria function and Fas splicing. (**a**) Representative picture of Mito-Tracker red labeled mitochondria in FAS-AS1 overexpressed cells or control cells. (**b**) Quantitative analysis of the representative picture’s fluorescence intensity. (**c**) Microstructure of mitochondria was observed by TEM in FAS-AS1 overexpressed cells or control cells. (**d**) Relative expression of sFas was determined by RT-qPCR 24 h after transfection with FAS-AS1 vector or control vector. (**e**) Relative expression of mFas was determined by RT-qPCR 24 h after transfection with FAS-AS1 vector or control vector. (**f**) The expression of several cell apoptosis signal regulators (cleaved-caspase 3, Bcl-2 and Bax) were examined by western blotting after transfection with FAS-AS1 vector or control vector. (**g**) The quantification of cleaved-caspase 3. (**h**) The quantification of Bax/Bcl-2. *P < 0.05, **P < 0.01.

We further analyzed how FAS-AS1 affected cell apoptosis. We used Mito-Tracker red CMXRos to label mitochondria and observed the mitochondria was more aggregated and fluorescence intensity was much brighter in FAS-AS1 overexpressed cells (Fig. 6a,b). Besides, we observed the morphology and structure of mitochondria using TEM. We found the mitochondrial densities were condensed, the cristae were diminished or even vanished, the mitochondria became swollen, crumpled and broken in FAS-AS1 overexpressed cell (Fig. 6c). As mentioned above, the GSEA analysis indicated genes regulated by FAS-AS1 was enriched in mRNA splicing. In light of the pivotal role of FAS-AS1 in regulating Fas splicing, we analyzed the two isoforms of Fas (sFas and mFas) in NPC cells transfected with FAS-AS1 vector. We revealed the relative expression of sFas isoform was significantly decreased in FAS-AS1 overexpressed cells, while the expression of mFas isoform was increased **(**Fig. 6d,e**)**. In addition, western blot was used to detect the effect of FAS-AS1 on apoptosis-related proteins and found that the expression of c-caspase3 and Bcl-2-associated X (Bax) were both significantly increased, while B-cell lymphoma-2 (Bcl-2) was decreased after FAS-AS1 overexpression **(**Fig. 6f–h**)**. These findings suggested that FAS-AS1 promoted NPC cells apoptosis by regulating mitochondria function, Fas alternative splicing and sequential activation of caspases 3.

## Discussion

In the era of precision medicine, individual’s genetic information is closely related to disease/healthy status and therapeutic effect^30–32^. Genetic predisposition has been widely believed to be a main risk factor for NPC pathogenesis. In the present study, we investigated the role of lncRNA FAS-AS1 and its genetic variation rs6586163 in NPC.

Over the past decades, researchers have identified a number of genetic loci that were associated with NPC, and the vast majority were located in protein coding genes^33,34^. It is of note that genetic variations in noncoding regions may also exert important regulatory functions in disease as the results from GWAS showed only 7% of diseases associated loci was located in protein-coding regions. Specifically, genetic variations in lncRNAs were able to affect the expression level, secondary structure or interfere the interaction between lncRNAs with their binding partners, thus affecting the disease occurrence and progression^35^. Previous study has reported lncRNA GAS5 rs2067079 (C > T) could affect the secondary structure of GAS5 and was associated with an obviously increased risk of radio-chemotherapy induced hematotoxicities in NPC patients^36^. In addition, linc00312 rs12497104 (G > A) could destroy the binding between mir-411-3p and linc00312, and affect the expression of linc00312, thus conferring increased susceptibility risk and poorer overall survival in NPC patients^29,37^.

In this study, we performed a case–control study to assess the association between FAS-AS1 SNPs and NPC susceptibility and prognosis. By applying some bioinformatics databases, we screened for SNPs with active regulatory elements in FAS-AS1 and finally focused on rs6586163. By retrieving PubMed, we found no one study has investigated the role of rs6586163 up to date. We demonstrated rs6586163 was correlated with decreased risk of NPC and better overall survival. We were very curious about how rs6586163 works. As predicted by the chromatin state at rs6586163, it was enriched with active transcription start sites. So, we performed luciferase reporter assay to confirm the role of rs6586163 in regulating transcriptional activity. Our results suggested rs6586163 might increase the transcriptional activity of FAS-AS1 and lead to aberrant expression of FAS-AS1 in NPC patients.

Disease associated genetic variation influence the transcription of lncRNA is not unique. A previous study has reported lncRNA PCAT19 rs11672691 suppressed binding of transcription factor NKX3.1 to the promoter of PCAT19-short and lead to prostate cancer progression^38^. Another study indicated lncRNA PCAT1 rs7463708 increased binding of transcription factor ONECUT2 to PCAT1 promoter, resulting in upregulation of PCAT1 and prostate transformation^39^. In addition to its role in regulating FAS-AS1 transcription, we analyzed the eQTL feature of rs6586163. We found rs6586163 was correlated with the expression of 36 genes, which were enriched in FAS signaling pathway, p53 pathway, apoptosis signaling pathway, and Wnt signaling pathway. These pathways were important in controlling cell apoptosis in cancer, indicating rs6586163 and FAS-AS1 might participate in NPC by regulating cell apoptosis.

Accumulating evidence has demonstrated that deregulation of FAS-AS1 contributed to the pathogenesis of diverse human disorders, including erythroid maturation^40^, systemic lupus erythematous^41^, renal transplant rejection^42^, neuropsychiatric disorders^43–45^, osteoarthritis^24^, and periodontitis^46^. However, studies about the role of FAS-AS1 in tumor is quite rare and the molecular function of FAS-AS1 in NPC is still unclear. We reveled FAS-AS1 was down-regulated in NPC and overexpression of FAS-AS1 was correlated with early clinical stage and better short-term treatment efficacy of NPC patients. Besides, the expression of FAS-AS1 was positively associated with Fas in NPC tissues. In vitro experiment showed FAS-AS1 could inhibit cell viability and promote apoptosis of NPC cells. By applying RNA-seq and GSEA analysis, we revealed FAS-AS1 might participate in mitochondrial regulation and contribute to cell apoptosis.

Mitochondria is the center of energy metabolism of the body that undergoes oxidative phosphorylation and synthesizes adenosine triphosphate (ATP). In addition, mitochondria play an essential role in calcium homeostasis and cell apoptosis^47^. Mitochondrial dysfunction can reduce the activity of respiratory chain enzymes, decrease the mitochondrial membrane potential, impede ATP synthesis, impair intracellular calcium homeostasis, resulting in mitochondrial permeability transition, intracellular fatty acid accumulation, oxidative stress increased, mitochondrial biosynthesis reduced, and finally leads to cell apoptosis or death^48^. Our result indicated overexpression of FAS-AS1 decreased the gene sets in mitochondrial translation, mitochondrial electron transport, mitochondrial respiratory chain complex I assembly, and metabolic process. The aberrant electron transfer in mitochondrial respiratory chain increases ROS production and oxidative stress, which could damage the mitochondrial structure and is the main cause of mitochondrial dysfunction. Excessive ROS can also induce the opening of mPTP and causing mitochondrial swelling. Swelling of mitochondria ultimately leads to the rupture of the outer membrane of mitochondria, releasing cytochrome C and apoptosis inducing factors, activating the caspase pathway and causing cell apoptosis or death^49^. The inner membrane of mitochondria is bent into folds known as cristae that house the protein components of the main energy-generating system of cells, which drives the phosphorylation of ADP to ATP and powers nearly all cellular activities^50^. By conducting TEM, we found the mitochondria became swollen, the cristae were reduced or even disappeared, and the membrane was ruptured in FAS-AS1 overexpressed cells. Moreover, we found the intracellular ATP content was decreased and the mitochondria morphology was aggregated in FAS-AS1 overexpressed cells. These findings provide the message that FAS-AS1 might promote cell apoptosis through regulating mitochondrial function.

To identify the key driver genes and possible mechanism of FAS-AS1 in mitochondrial dysfunction, we constructed a PPI network and performed hub gene analysis of FAS-AS1 regulated DEGs. The top 5 hub genes were HSP90AA1, CS, BCL2L1, SOD2, and PPARGC1A. Evidence has proved that HSP90AA1 potentiates the glycolysis and proliferation, reduces the apoptosis and mitochondria respiration of cells^51^. Acting as a rate-limiting enzyme in the citrate cycle, CS is a key regulators of mitochondrial lipid metabolism and regulates the fatty acid activation. CS has been reported to be upregulated in a variety of cancers and associated with aggressive progression and poor prognosis^52^. BCL2L1 is a mitochondrial apoptotic regulator that regulates outer mitochondrial membrane channel opening, mitochondrial membrane potential, and controls the production of reactive oxygen species and release of cytochrome C, both of which are the key inducers of cell apoptosis^53^. SOD2 is an enzyme responsible for reducing superoxide radicals in mitochondria^54^. PPARGC1A acts as a transcriptional coactivator that regulates the genes involved in mitochondrial respiratory chain and drives virtually all aspects of mitochondrial function and biogenesis^55^. The expression of PPARGC1A is positively correlated with mtDNA copy number, and knockdown of PPARGC1A reduces the mitochondrial content and decreases cell proliferation^56^. These hub genes may provide some new insights for the potential regulating mechanism of FAS-AS1 in mitochondrial dysfunction.

FAS-AS1 is an antisense lncRNA transcribed from the opposite strand of Fas. One way that antisense lncRNAs exert its function is through cis regulation of sense transcript or genes located in their vicinity^57^. A number of studies have reported the role of FAS-AS1 in regulating Fas related apoptosis. sFas is an alternative splice product of Fas pre-mRNA, commonly created by exclusion of exon 6 (FasΔEx6) that encode a transmembrane spanning sequence^58^. Neoplastic cells are frequently resistant to Fas-mediated apoptosis, evade Fas signals through down regulation of Fas and produce soluble Fas (sFas) proteins that bind FasL thereby blocking apoptosis^59^. Our result also confirmed FAS-AS1decreased sFas/mFas isoform ratio in NPC cells, leading to increased apoptotic cells.

Previous studies also indicated the levels of FAS-AS1 was inversely correlated with production of sFas in breast cancer and B-cell lymphoma^19,21^. In addition, the serum levels of sFas were elevated in patients with malignant lymphoma and chronic lymphocytic leukemia (CLL)^60^. The overall survival and disease-free survival rates are significantly lower in lymphoma patients with elevated serum sFas levels^61^.

**Figure 7:**
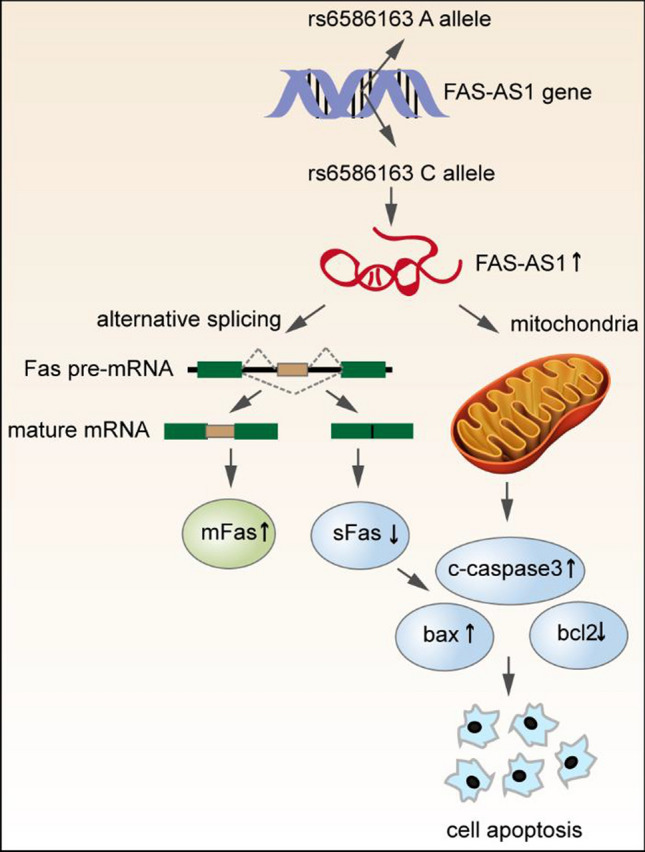
The regulatory mechanism of rs6586163 and FAS-AS1 in NPC. FAS-AS1 rs6586163 increases the transcriptional activity of FAS-AS1 and contributed to ectopic overexpression of FAS-AS1 in NPC. Overexpression of FAS-AS1 induces apoptosis by regulating mitochondrial function and Fas alternative splicing, which is followed by activation of caspases 3, up-regulation of bax and down-regulation of bcl2.

In conclusion, we demonstrated FAS-AS1 and its genetic variation rs6586163 were associated with NPC occurrence and prognosis. rs6586163 might have a protective role in NPC through influencing the structure of FAS-AS1, increasing the transcriptional activity and expression of FAS-AS1. FAS-AS1 was downregulated in NPC and overexpression of FAS-AS1 suppressed NPC cell proliferation and promoted cell apoptosis possibility via regulating mitochondrial function and Fas splicing (Fig. 7). Our findings may be helpful in understanding the function of FAS-AS1 in NPC carcinogenesis and searching for new diagnosis biomarker and potential therapeutic target for NPC.

## Supplementary Information

Supplementary Tables.

Supplementary Figures.

## Data Availability

The datasets generated during and/or analysed during the current study are available in the Supplementary Dataset File and Gene Expression Omnibus, GSE225264 (https://www.ncbi.nlm.nih.gov/geo/query/acc.cgi?acc=GSE225264). The secure token number “sryvaggylnunnmr” is created to allow review of record GSE225264.
