# Supplementary figures and images for: LncRNA FAS-AS1 upregulated by its genetic variation rs6586163 promotes cell apoptosis in nasopharyngeal carcinoma through regulating mitochondria function and Fas splicing
Source: Sci Rep. 2023 May 22;13:8218. doi: 10.1038/s41598-023-35502-z (PMC10203136; doi:10.1038/s41598-023-35502-z)

Blots-actin

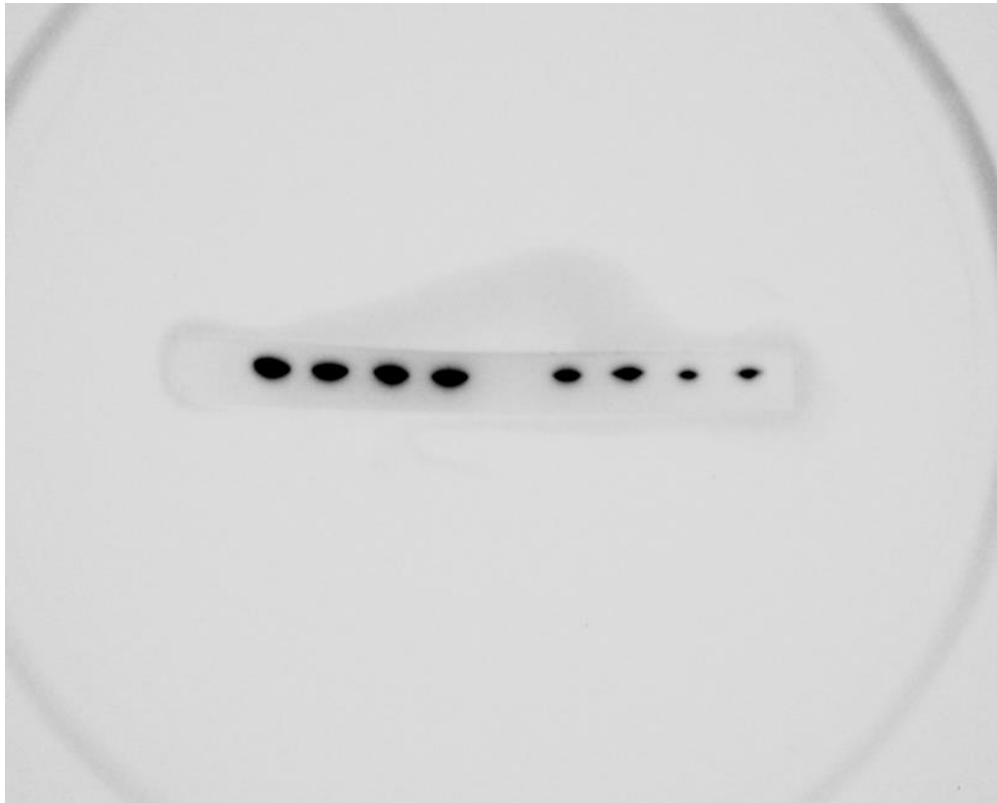

Blots-bax

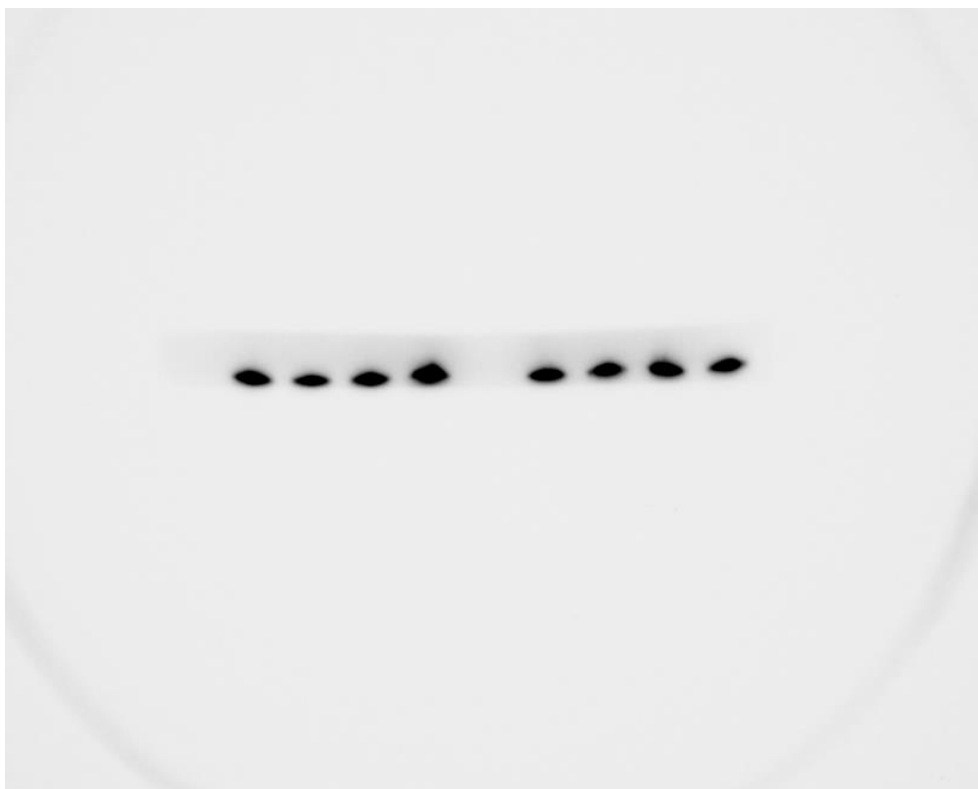

Blots-bcl2

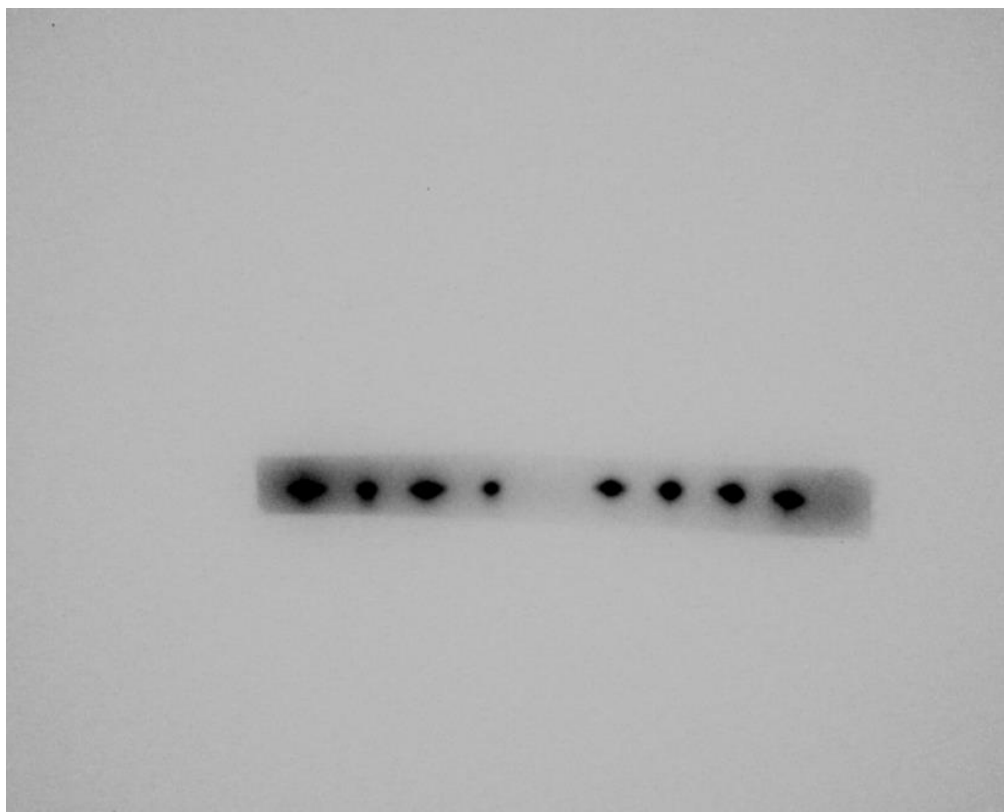

Blots-cleaved caspase3

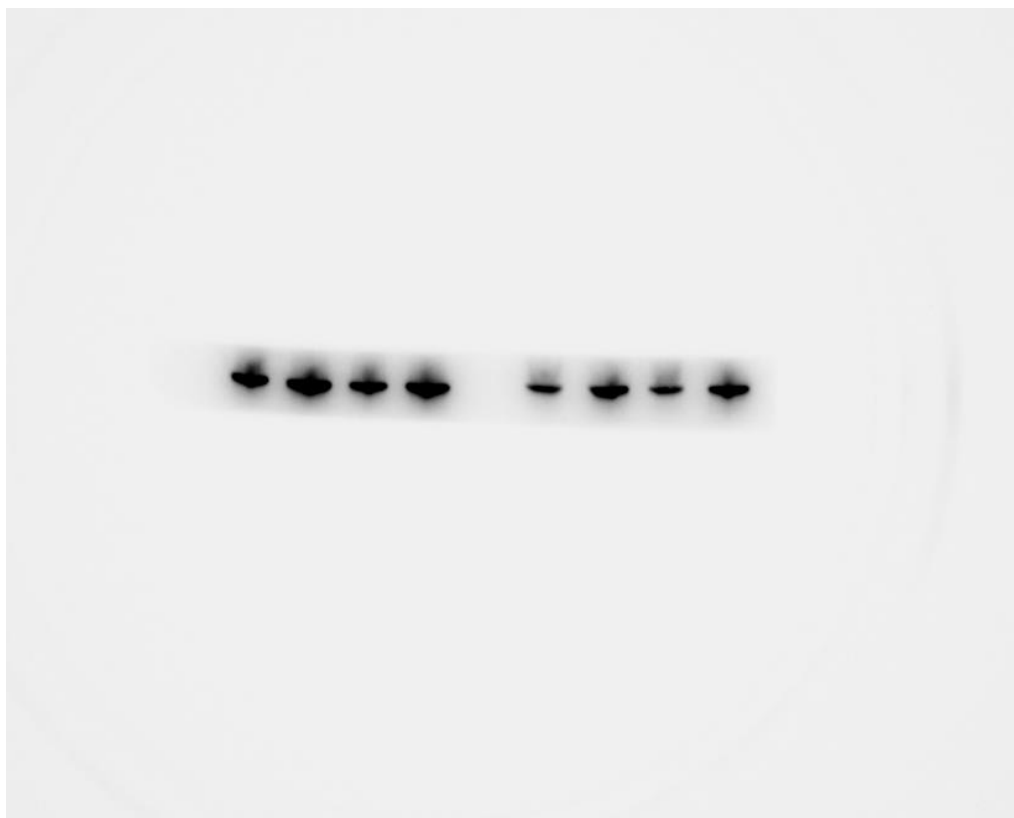

Supplement: Supplementary file 2 — Supplementary Figures. [file 41598_2023_35502_MOESM2_ESM.pdf]
